# Physical activity telephone coaching intervention for insufficiently physically active ambulatory hospital patients: Economic evaluation of the Healthy 4U-2 randomised controlled trial

**DOI:** 10.1371/journal.pone.0270211

**Published:** 2022-06-23

**Authors:** Stephen Barrett, Stephen Begg, Paul O’Halloran, Christopher M. Doran, Michael Kingsley

**Affiliations:** 1 Health Promotion Department, Bendigo Health Care Group, Bendigo, Victoria, Australia; 2 La Trobe Rural Health School, La Trobe University, Bendigo, Victoria, Australia; 3 School of Psychology and Public Health, La Trobe University, Bundoora, Victoria, Australia; 4 Centre for Resilience and Wellbeing, Central Queensland University, Queensland, Australia; 5 Holsworth Research Initiative, La Trobe Rural Health School, La Trobe University, Bendigo, Victoria, Australia; 6 Department of Exercise Sciences, University of Auckland, Newmarket, New Zealand; Prince Sattam Bin Abdulaziz University, College of Applied Medical Sciences, SAUDI ARABIA

## Abstract

The Healthy 4U-2 randomised controlled trial demonstrated that a physical activity (PA) telephone coaching intervention was effective for improving objectively-measured PA and health-related outcomes. The current study reports on an economic evaluation performed alongside the trial to determine whether this effective intervention is also cost-effective from a healthcare funder perspective. Participants (N = 120) were insufficiently physically active adults recruited from an ambulatory care clinic in a public hospital in regional Australia. The primary outcome was change in moderate-to-vigorous physical activity (MVPA) measured using accelerometers. Changes in quality-adjusted life-years (QALYs) were derived from the 12-Item Short Form Health Survey Questionnaire (SF-12). Incremental cost-effectiveness ratios (ICERs) were calculated for each outcome. Uncertainty of cost-effectiveness results were estimated using non-parametric bootstrapping techniques and sensitivity analyses. The mean intervention cost was $132 per person. The control group incurred higher overall costs compared to intervention ($2,465 vs. $1,743, respectively). Relative to control, the intervention resulted in incremental improvements in MVPA and QALYs and was deemed cost-effective. Probabilistic sensitivity analysis indicated that compared to control, the intervention would be cost-effective for improving MVPA and QALYs at very low willingness to pay thresholds. Sensitivity analyses indicated that results were robust to varied assumptions. This study shows that PA telephone coaching was a low-cost strategy for increasing MVPA and QALYs in insufficiently active ambulatory hospital patients. Findings of health benefits and overall cost-savings are uncommon and PA telephone coaching offers a potentially cost-effective investment to produce important public health outcomes.

## Introduction

Insufficient physical activity (PA) is an established risk factor for the development of a number of chronic diseases such as coronary heart disease, stroke, cancer and type 2 diabetes [1]. Insufficient PA also imposes a serious financial burden on society as a result of morbidity, healthcare costs and productivity loss [2]. The direct cost of insufficient PA on healthcare systems worldwide is estimated to be 53.8 billion international dollars [3]. The combined health and economic burden that insufficient PA exacts on society highlights the need for interventions to stimulate individuals to increase and maintain PA, which might improve public health outcomes and ultimately reduce the associated healthcare costs [4].

Individually tailored behaviour change interventions are required to promote PA change [5]. Key requirements for PA behaviour change include increasing motivation, self-efficacy, effective goal setting, lapse prevention and performance feedback [6]. Telephone coaching is an established method of delivering individually tailored PA behaviour change interventions [5]. Telephone coaching enables personal relationships to develop and repeated contacts provides opportunity to address the key requirements for PA change [6].

To address the burden of insufficient PA, all sectors of the healthcare industry need to incorporate strategies to promotion PA [7]. Ambulatory hospital clinics are important settings to promote PA change [8]. The health of hospital patients is likely to be compromised and patients can be motivated to consider behaviour change [9]. Doctors are repeatedly seen as reliable sources of health information and doctors’ recommendations to increase PA can influence change [10]. We conducted a randomised controlled trial that used surgeons consulting in ambulatory hospital clinics to verbally recommend patients to increase PA and engage in a follow-on PA telephone coaching intervention. The surgeons highlighted the need for PA change, and signposted patient to the PA coaching intervention designed to address determinants of PA change. The results of the trial indicated that the PA telephone coaching resulted in significant maintained improvements in device-measured PA and health-related outcomes [11, 12]. The cost to improve these outcomes has not been reported.

Despite the increased use of PA coaching interventions, economic analyses of intervention studies are relatively scarce. A recent systematic review found only 9 studies reported the cost-effectiveness of PA coaching to increase PA in adult populations [13]. None of these interventions were delivered to hospital populations [13]. Economic analyses are essential for bridging the research-to-practice gap and are needed to inform implementation feasibility [14]. Due to fiscal constraints, hospitals and healthcare decision makers need to be informed of interventions that provide value for money. The absence of economic evidence might hamper potential implementation of PA telephone coaching research into healthcare policy and practice [4].

The purpose of the current study was to evaluate the cost-effectiveness of a PA telephone coaching program for increasing device-measured PA and quality-adjusted life-years (QALYs) in insufficiently physically active ambulatory hospital patients from a healthcare funder perspective.

## Methods

This study is an economic analysis performed alongside the Healthy 4U-2 randomised controlled trial and was reported in line with the Consolidated Health Economic Evaluation Reporting Standards (CHEERS) reporting guidelines [15]; see S1 Checklist (CHEERS Checklist).

**Fig 1:**
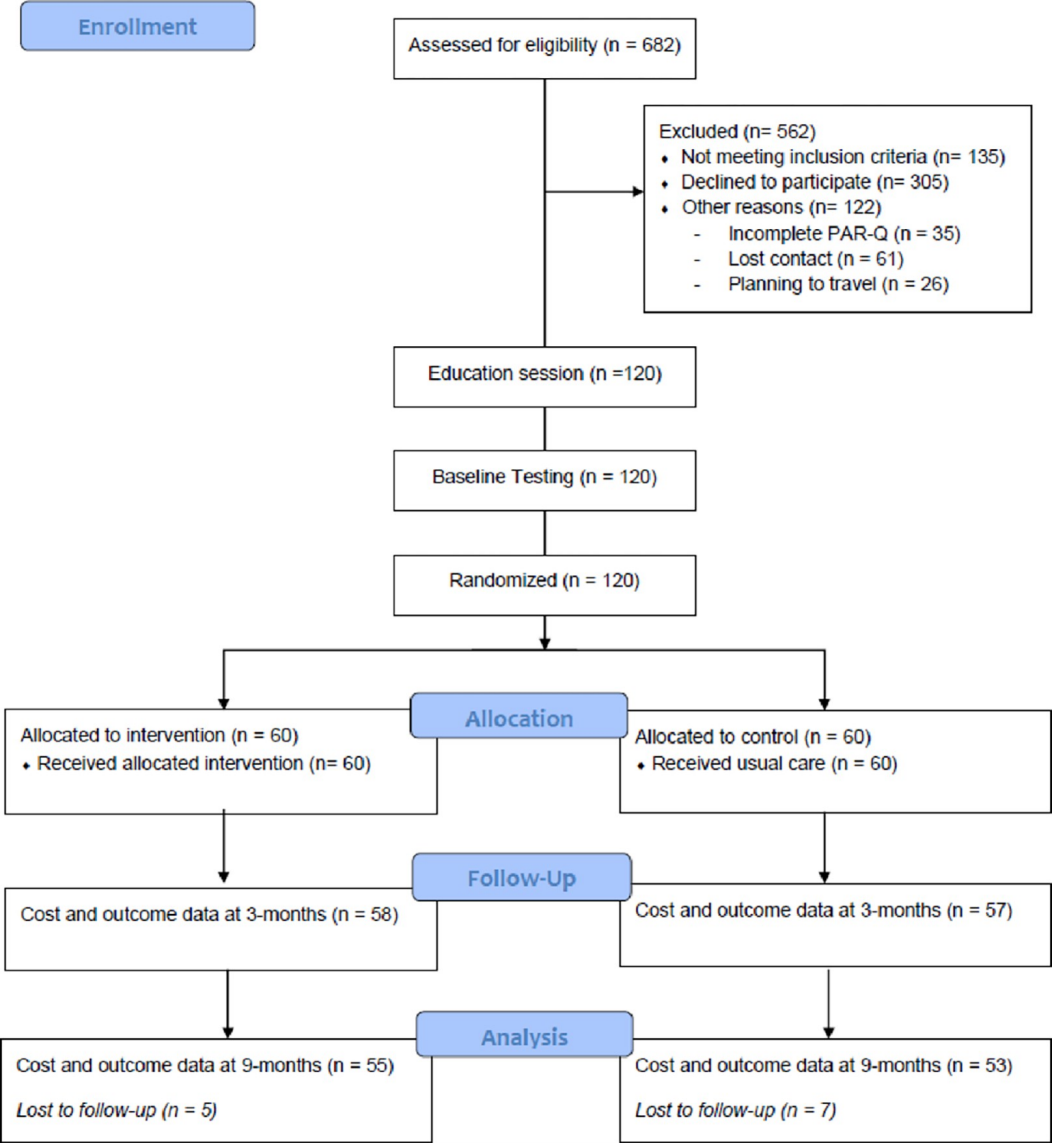
CONSORT flow diagram.

The Healthy 4U-2 study design, participants, intervention, outcomes have been described in detail elsewhere [11]. In brief, from January 2019 to September 2019, a total of 120 insufficiently active adults were recruited from ambulatory medical clinics at a major hospital in a regional town in Victoria, Australia into a PA coaching intervention study. During ambulatory medical consultations surgeons provided patients with a verbal recommendation to engage in PA coaching and the research flier. The participant flow through the study is depicted in Fig 1.

The study was approved by the Research Ethics Committees of Bendigo Health Care Group (approved November 1, 2018; reference number LNR/18/BHCG/44121) and La Trobe University College of Science Health and Engineering Human Ethics Sub-Committee (approved November 13, 2018). Participants provided informed written consent prior to starting the study. The study was registered with the Australian and New Zealand Clinical Trials Registry (ACTRN12619000036112) prior to participant recruitment. The study protocol and supporting CHEERS checklist for this trial are available as supporting information; see S1 File (Study Protocol) and S2 Checklist (CONSORT Checklist).

### Intervention

All enrolled participants attended a 30-min group education session prior to group allocation. The education session was a facilitated learning session carried out using a self-determination theory framework [16]. Self-determination theory was used to support, educate and motivate participants around PA change and PA self-management [16].

Participants in the intervention arm completed an individually-delivered telephone-based PA coaching intervention that comprised integrated motivational interviewing and cognitive behaviour therapy (MI-CBT). The coaching was delivered in five 20-min sessions over 12 weeks. The intervention was delivered by an experienced Australian Health Practitioner Regulating Authority registered physiotherapist trained in MI-CBT including its application in research [17]. The clinician delivered the intervention sessions as part of a stand-alone research project and not in addition to existing clinical time. Participants enrolled into the control arm attended the education session only.

### Measurement of effects

Outcome measures were recorded at baseline, after 3 months (post-intervention) and at 9 months (follow-up) by assessors blinded to the study group assignment (Fig 1). The primary outcome measure was change in minutes per day of moderate-to-vigorous physical activity (MVPA) measured by accelerometry (wGT3X-BT; Actigraph, USA). Daily measures of MVPA were determined using the manufacturer’s software (Actilife; Actigraph, USA) and the Freedson Adult (1998) cut point (vector magnitude >1961 cpm) [18]. A minimum wear time of ≥10 hour/day for 5 of the 7-day period, including at least one weekend day was required to be included in the analysis.

Weekly PA were summed from using daily totals for persons with 7 valid days of monitoring, or estimated as seven times the mean daily total for persons with 5–6 valid days of monitoring. The summed weekly totals were used to classify participants as either meeting the recommended PA guidelines (≥150 mins/week of MVPA), or not [19].

Health-related quality of life (HrQoL) in terms of QALYs was assessed using the 12-Item Short Form Survey (SF-12) [20]. The SF-12 scores were converted to utility scores using the standard Brazier algorithm [21]. Utility scores were measured on a scale of 0–1 where a higher score indicated a more favourable health state. The utility estimates were converted to QALYs by calculating the ‘area under the curve’ utility estimates for the different follow-up time intervals for each participant, weighted by the length of follow-up at that time interval.

### Measurement of costs

The economic evaluation was performed from the perspective of the healthcare funder. Healthcare costs, community service costs and intervention costs were collected over the trial period. All costs were calculated in 2019 Australian dollars (AU$). The costs and time attributable to undertaking the research and the development of the intervention were excluded, as these costs will not have to be paid in future implementation.

The costs relevant for implementation of the intervention included invitation costs, printing and postage costs, education session delivery time, intervention delivery time and research assistant time. We calculated the education group facilitator’s time at 2.5 hours for each group. This included 1.0 hour to prepare for the group meeting (review of educational material), 0.5 hours to facilitate the group meeting itself, and 1.0 hour was allocated to set-up and clean up after the group meeting. The education group facilitator’s cost per participant was calculated by dividing the facilitator’s cost per meeting by the number of participants who attended the meeting. The costs for the individually delivered intervention were calculated as the time spent in delivering the PA telephone coaching with the participants. The education group and intervention were delivered by a senior allied health clinician; the time was valued at an annual salary of AU$84, 168 using the national mean registered allied health professional salary [22]. The intervention assistant’s time was spent reviewing symptom reports, accelerometer problems, voicemails left by participants and undertaking reminder phone calls to participants. This time was valued at using the annual salary of an Allied Health Assistant (AU$46, 758) [22].

Based on economic evaluation methodology we deemed the following health care costs to be relevant: consultations with general practitioner, allied health clinician (e.g., dietician, physiotherapist), practice nurse, medical specialist, or any remaining health care providers [14]. We included hospital admissions as relevant costs. Healthcare costs were retrospectively assessed at 3-month intervals over the 9-month follow-up period using a costing questionnaire. To assess healthcare costs, participants reported consultations with health care providers and the number of times they used the service. Where appropriate, participants reported hospital admissions, including surgeries and the number of nights they stayed in the hospital. Healthcare costs were valued using National Weighted Activity Unit calculators from the Australian Independent Hospital Pricing Authority [23]. Unit pricing for subcategories of healthcare use is provided in S1 Table (Unit pricing for subcategories of healthcare use).

### Sample size and power

The sample size calculation was based on findings from a comparable trial with a relevant population of insufficiently physically active ambulatory hospital patients [17]. Based upon this data a sample size of 50 participants per treatment group was required to detect a between group difference of 30 ± 13 (mean ± SD) mins/week MVPA, with the alpha set at 0.05, and the power set at 0.80. To protect for an assumed 20% loss to follow-up, a total of 120 participants were recruited.

### Statistical analysis

The mean ± SD for the overall cost and for the change in each outcome at 9 months was summed. The incremental cost-effectiveness ratio (ICER) was calculated for all outcomes by dividing the difference in costs by the difference in effects between the intervention and control groups. The difference in effects between the two groups was calculated using a change from baseline approach to control for different baseline utilities. To account for uncertainty in the ICER estimates, cost and effects pairs were bootstrapped (1000 bootstrap replications). Results of the bootstrapping analysis computed a cost-effectiveness acceptability curve (CEAC) and cost-effectiveness plane for each outcome. The CEAC indicates the probability that the intervention was cost effective at different values of willingness to pay for the additional improvement in the outcome [24]. The cost-effectiveness plane visually represents the differences in costs and health outcomes between treatment alternatives on a graph. The graph demonstrates both the uncertainty and the magnitude of the estimates [14].

If an intervention results in higher effects with lower costs the intervention is preferred (dominant); conversely, an intervention with lower effects and higher costs is not preferred (dominated) [14]. Where interventions results in higher effects and higher costs, or lower effects and lower costs, the preference for an intervention condition depends on how much the stakeholder is willing to pay for an incremental gain in the outcome [14]. At present there is no fixed willingness-to-pay (WTP) threshold for PA outcomes. Preventive health interventions often use a maximum WTP of AU$30,000/QALY [25].

To account for uncertainty of parameter estimates, sensitivity analyses were performed to examine how the results changed under different input assumptions. In the first two analyses, program costs were varied by 20% in either direction before recalculating the ICERs. The 20% variation in program costs can provide an indication of the cost effectiveness of the intervention using clinicians across differing pay grades and might be of interest to healthcare funders. The third sensitivity analysis used the summed weekly MVPA totals to estimate the ICER for each additional minute of MVPA per week. The fourth sensitivity analysis used the summed weekly MVPA totals to estimate the ICER for changing one individual from insufficiently physically active to sufficiently physically active [19]. The fifth sensitivity analysis was performed considering outcomes at 12 months. To calculate this, health service utilisation was calculated from participants using the costing questionnaire; outcome data was extrapolated using the last-observation carried forward method [26].

IBM SPSS version 27.0 (SPSS Inc., Chicago, IL, USA) and Microsoft Excel were used in the data analyses. The University Of Oxford Applied Methods Of Cost-effectiveness Analysis in Healthcare bootstrapping models [27] were used in Microsoft Excel.

## Results

In total, 120 participants were recruited into the study. The group consisted of 81 females (68%) and 39 males, with a mean age of 53 ± 8 years. No statistically significant differences were observed in demographic and clinical characteristics between the intervention and control groups at baseline. More than half (52%) of participants were classified as obese and 72% were in gainful employment. The mean group participation time was 32 ± 5 min, and the mean participant time spent in the intervention was 90 ± 12 min. The programme resources and cost per participant are described in Table 1 with the PA telephone coaching cost representing 66% of the total cost.

**Table 1:** Healthy 4U-2 programme delivery costs.

Item	Provider	Units	Time (hours)	Cost/hour AU$	Total AU$ cost per participant
**Intervention group**					
Group Sessions	AHP	1	2.5	42.60	12.50
Phone call reminders	AHA	5	0.2	23.70	23.70
Intervention sessions	AHP	5	0.30	42.60	63.90
Program manual					2.50
**Total cost/participant**					**132**
**Control group**					
Group Sessions	AHP	1	2.5	42.60	12.5
Program manual					2.5
**Total cost/participant**					**15**

AHA: Allied Health Assistant; AHP: Allied Health Professional.

### Healthcare resource utilisation and cost

Total healthcare utilisation costs after 9 months for the intervention group were AU$1,743 compared to AU$2,465 for the control group, representing a statistically significant difference in mean healthcare resource utilisation cost between the groups. The differences in subcategories of healthcare resource utilisation cost between the groups are presented in Table 2. Compared to the intervention group there was a higher mean healthcare resource utilisation cost in the control group for general practitioner, physiotherapy, medical specialist consultations and for hospital admissions. The biggest difference in healthcare resource utilisation costs between the two groups was the higher costs associated with hospital admissions in the control group compared to the intervention group.

**Table 2:** Total program and healthcare resource costs.

Cost category	Unit cost price AU$	Intervention AU$ Cost (SD)	Control AU$ Cost (SD)	P-value ^a^
Program cost	-	132	15	
Health care cost	-	1611 (1705)	2465 (3355)	0.031
General Practitioner	108.9 ^b^	203 (116)	338 (159)	0.005
Practice Nurse	59.35 ^b^	81 (139)	32 (53)	0.009
Physiotherapy	149 ^b^	67 (99)	159 (198)	0.002
Exercise Physiology	138 ^b^	90 (129)	67 (116)	0.362
Allied Health	Varying ^b^	263 (272)	224 (286)	0.451
Medical Specialist	Varying ^b^	128 (177)	301 (350)	0.001
Hospital admission	Varying ^b^	681 (1588)	1115 (1943)	0.142
Emergency Department	Varying ^b^	99 (192)	142 (250)	0.391
**Total cost**		1743 (1705)	2465 (3356)	0.042

^a^ t-test between intervention and control groups

^b^ Unit cost prices are indexed based on Australian data

#### Cost-effectiveness

The cost-effectiveness analysis demonstrated that for PA change the intervention group dominated the control group (Table 3). Participants in the intervention group increased their average MVPA by 12 mins/day with a cost saving of AU$722 per participant over the 9-month follow up compared to control group. The CEAC indicated that the intervention was preferred over the control group for all WTP thresholds, with a 99% probability of the intervention being cost-effective compared to control (see S1 Fig cost-effectiveness acceptability curves). The distribution of the incremental cost-effect pairs from the 1,000 bootstrap replications are shown in Fig 1. In 100% of the replications the PA telephone coaching arm produced greater MVPA change at a lower cost than control.

**Table 3:** Costs, changes in outcomes and incremental cost-effectiveness ratios at follow-up.

Outcome	Cost AU$/Participant	Outcome	Δ cost AU$	Δ outcome	ICER
**MVPA**					
Control	2465	10			
Intervention	1743	22	-722	12	AU$ -61/min MVPA per day ^a^
**QALYs**					
Control	2465	-0.006			
Intervention	1743	0.007	-722	0.015	AU$ -48,133/QALY ^a^

ICER: incremental cost-effectiveness ratio; MVPA: moderate-to-vigorous physical activity; QALYs: quality-adjusted life-years

^a^ Dominant

#### Cost-utility

**Fig 2:**
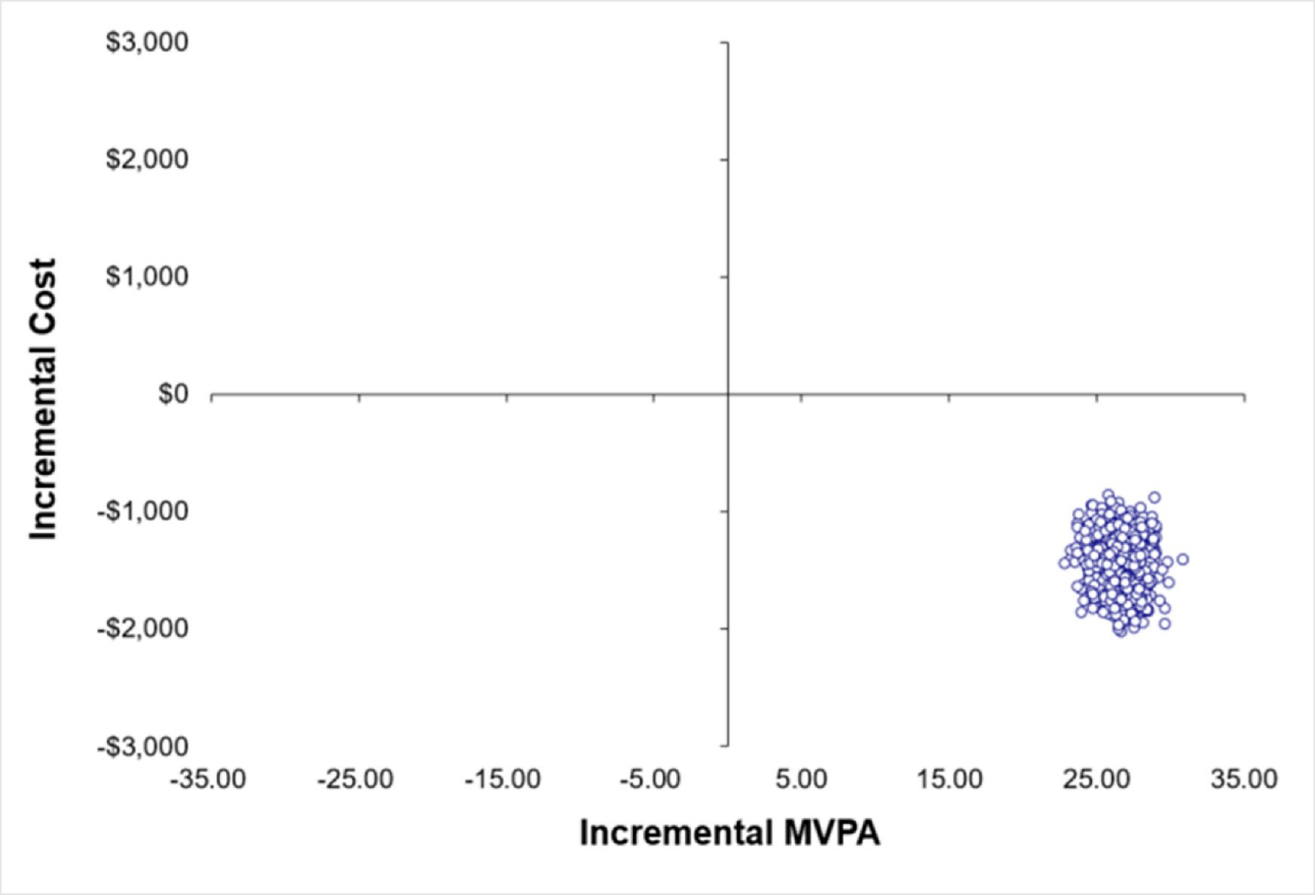
Incremental cost-effect pairs from 1000 bootstrap resamples for MVPA change.

**Fig 3:**
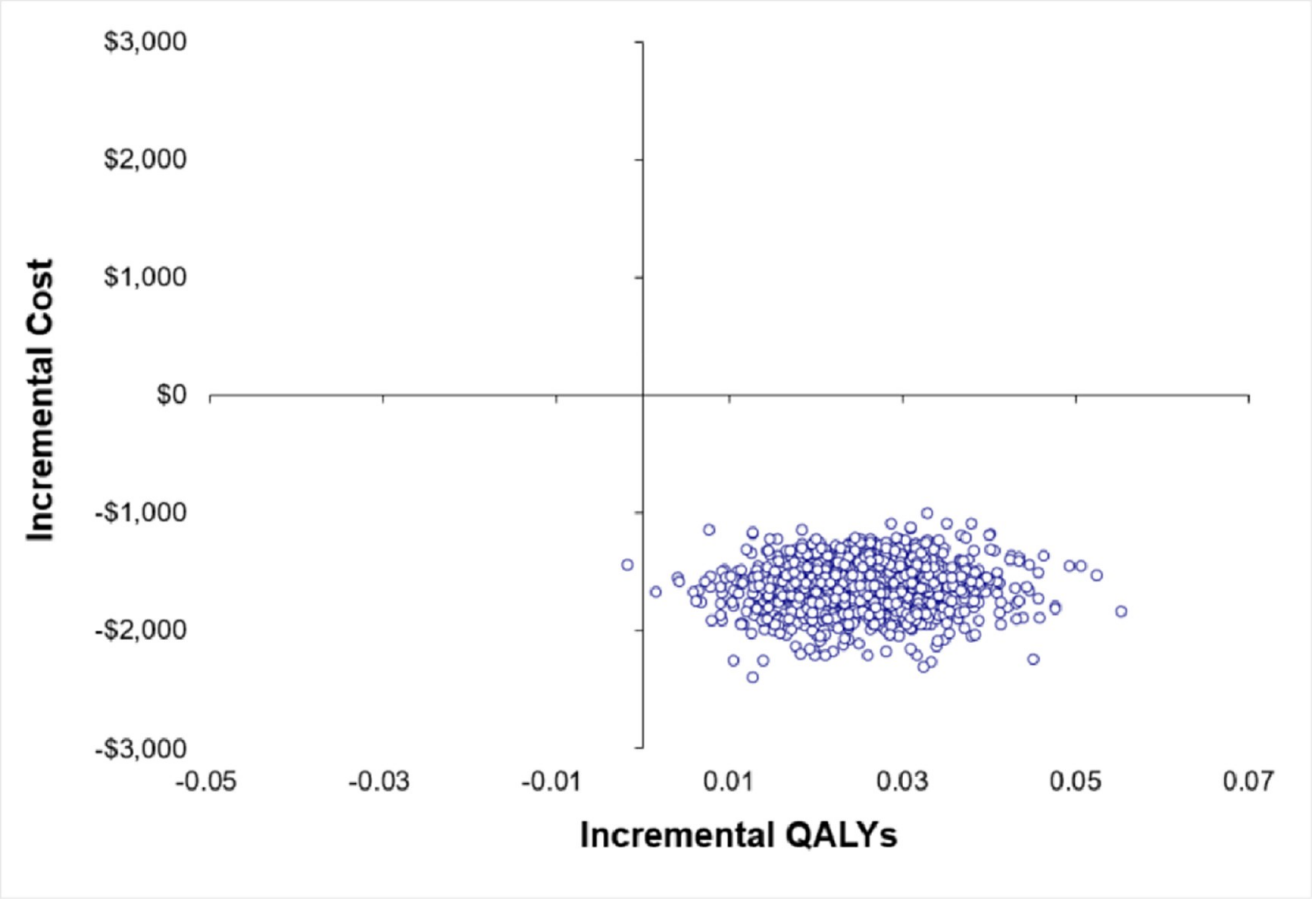
Incremental cost-effect pairs from 1000 bootstrap resamples for QALY change.

Participants who received the PA telephone coaching intervention gained 0.015 more QALYs than the control group over the follow-up period (Table 3). The PA coaching intervention dominated the control group, producing the increased QALYs at lower overall costs. The intervention would be considered cost-effective for all WTP thresholds (see S1 Fig cost-effectiveness acceptability curves). Figs 2 and 3 highlight that in 99% of the bootstrapped incremental cost-effect pairs the PA coaching arm resulted in a higher PA and QALY gains than the control arm at a lower cost.

### Sensitivity analyses

In the sensitivity analyses, the PA telephone coaching was the dominant intervention across all of the individual comparisons. When program implementation costs were varied 20% in each direction the PA telephone coaching resulted in improved outcomes and lower overall costs (Table 4). The third and fourth sensitivity analyses demonstrated that relative to control the PA telephone coaching intervention cost less to increase each additional minute of MVPA per week, and to attain sufficient PA to meet the recommended PA guidelines at follow-up. When the HrQoL findings were extended to 12 months the incremental cost difference between the groups decreased slightly, while the incremental QALY gain increased. The PA telephone coaching was dominant in this analysis, resulting in increases in QALYs at lower costs to control.

**Table 4:** Sensitivity analyses for costs, changes in outcomes and incremental cost-effectiveness ratios at follow-up.

Outcome	Cost AU$/Participant	Effect	Incremental cost AU$	Incremental effect	ICER
**MVPA +20% variation in cost**					
Control	2958	10			
Intervention	2092	22	-893	12	AU$ -76/min MVPA per day ^a^
**MVPA -20% variation in cost**					
Control	1972	10			
Intervention	1395	22	-577	12	AU$ -49/min MVPA per day ^a^
**QALY +20% variation in cost**					
Control	2958	-0.007			
Intervention	2092	0.008	-893	0.015	AU$ -57,733 QALY ^a^
**QALY -20% variation in cost**					
Control	1972	-0.006			
Intervention	1395	0.007	-577	0.015	AU$ -38,478 QALY ^a^
**MVPA min per week**					
Control	2465	72			
Intervention	1743	157	-722	85	AU$ -8.49/min MVPA per week ^a^
**PA guidelines attained**					
Control	2465	2/60 (3%)			
Intervention	1743	30/60 (50%)	-722	47%	AU$ -1,536/PA guideline achieved ^a^
**QALY at 12 month follow-up**					
Control	2510	-0.017			
Intervention	1822	0.018	-688	0.035	AU$ -19,657 QALY ^a^

ICER: incremental cost-effectiveness ratio; MVPA: moderate-to-vigorous physical activity; QALYs: quality-adjusted life-years

^a^ Dominant

## Discussion

This study examined the clinical and economic implications of a PA telephone coaching intervention for changes in PA and QALYs for insufficiently physically active ambulatory hospital patients. The economic analyses demonstrated that for all outcomes the PA telephone coaching intervention dominated the control group, resulting in improved outcomes at a lower overall cost. To the best of our knowledge, this is the first study to evaluate the cost-effectiveness and cost-utility of a PA telephone coaching program for ambulatory hospital patients from a healthcare funder perspective. The inclusion of healthcare resource utilisation and costs in our calculations provides a more ecologically valid picture of costs associated with the integration of preventive health into routine hospital care.

The Healthy 4U-2 program was relatively low cost at AU$132 per participant and resulted in the average attainment of 22 ± 10 min of MVPA per day at the 9-month follow-up. Costs of PA coaching interventions vary. Intervention costs have been reported at greater than US$300 per person [13, 28], between US$100 and US$250 per person [17, 29], and 1 study reported intervention costs lower than US$100 per person [30]. Only 1 study was carried out with ambulatory hospital patients [17]. Hospitals are encouraged to deliver effective and efficient prevention programs targeting high-risk individuals to increase PA and promote individual self-management [31]. Despite this encouragement there is a scarcity of evidence relating to the economic implications of adding preventive interventions to routine hospital care where implementing an intervention requires an upfront investment of money.

The Healthy 4U-2 program was cost-effective as it resulted in lower healthcare funder costs and an increase in daily MVPA compared to the control group. Although the intervention required an upfront investment, the cost of $132 per participant is very low relative to the cost of a hospital admission. In addition, within 9 months this investment resulted in cost savings to the healthcare system due to decreased healthcare costs. Changes in PA have been shown to decrease healthcare costs within a 2-year period [32]. The increase in PA and decreased healthcare utilisation resulted in lower overall costs and explain the high probability of the PA telephone coaching being cost-effective. At present there is no standard value for how much society or healthcare funders are willing to pay per additional minute of MVPA or any incremental change in PA, but the negative ICER indicates that compared to control the PA telephone coaching intervention represents an effective strategy for increasing PA among ambulatory hospital patients at very low WTP thresholds. This study provides robust data allowing healthcare services to consider whether they are willing to pay AU$132 to have individuals undertake 22 mins MVPA per day at 9 months and decrease healthcare utilisation costs, or if they would allocate that money to other services.

A number of reviews have investigated the cost-effectiveness of interventions aiming to increase PA [13, 33]. The standardised PA effect measure used in these reviews was the metabolic equivalent of task (MET) measured in MET-hours gained per person per day. Using the formula by Wu et al. [33] the Healthy 4U-2 intervention resulted in 0.9 MET-hours gained per person per day. This is relatively strong incremental PA gain that can lead to substantial health benefits for inactive populations as the largest health gains are derived in the first 15–29 mins/day of PA by insufficiently active individuals [34]. The gain of 0.9 MET-hours/day is comparable to the 0.84 [35, 36] and greater than the 0.35 [30] and 0.26 MET-hours gained [37] in similar PA coaching interventions. Only 1 of these studies used accelerometers to measure PA [30]. Higher effects are generally observed in studies using subjective PA measures compared to device measured [33], which strengthens the confidence in the cost-effectiveness of our intervention.

In the cost-utility analysis the negative ICER indicates that relative to control the PA telephone coaching intervention represents an effective strategy for increasing QALYs among ambulatory hospital patients. The negative ICER falls far below the commonly used threshold of AU$30,000/QALY gained used in preventive health interventions. Physical activity coaching interventions are typically used to modify specific factors that can predispose individuals to risk of chronic disease over the longer term [38]. The long-term impact of PA coaching interventions on health-related quality of life is less established. The 0.015 incremental change in QALYs observed in the Healthy 4U-2 study was similar to incremental change of 0.01 QALYs [37] and greater than the 0.009 [39] and 0.007 [17] QALYs found in similar studies. Ewald et al. [36] found that telephone coaching had no impact on QALYs. Our current study was the only one in which the intervention dominated the control for QALYs gained. The change in QALYs over 9 months was a combination of the 0.006 decrease in the control group and the 0.007 increase in the intervention group. Extrapolating the findings to 12 months in the sensitivity analysis, the QALYs gained from the area under the curve increased to 0.035, driven by a -0.017 fall in the control group and an increase of 0.018 in the intervention. The changes in QALYs support the hypothesis that a key impact of behaviour change interventions on quality-of-life might be to attenuate expected declines in HrQoL over time [40]. Integrating PA telephone coaching intervention into ambulatory hospital care represents a low-cost strategy to simultaneously increase PA and health-related quality-of-life.

This study was unique in that we used consulting hospital surgeons to identify insufficiently physically active individuals and then recommend that they engage with the PA telephone coaching service. This approach was based on hospital surgeons’ stated preferences [41] and represented a simulation of integrating brief preventative health interventions into secondary care. The PA telephone coaching intervention was carried out in addition to standard care, not as a substitution. The addition of preventive health interventions are likely to cost hospitals more upfront, demonstrating the importance of investigating value for money. There is a scarcity of economic evaluations of telephone coaching carried out in real-life settings using an RCT design, and this study offers healthcare providers an estimate of the costs and effects of adding a preventative health intervention to clinical care. This economic evaluation was nested within a strong randomised controlled trial study design including long-term observations and a broad costing perspective, which provides a robust description of costs and benefits [14].

This study has a number of limitations. First, participant time costs were not accounted for in the costing analysis to provide a more societal perspective. The intervention was delivered in 5 x 20-min sessions via the telephone and required relatively small amounts of participant time. As such the opportunity costs to participants were expected to be small and were not included in the analyses. Second, the sample size and power calculations were based upon expected effect size for PA change. Estimating sample size and power are common challenges in trials evaluating both clinical and cost-effectiveness outcomes [42]. Based upon recommendations for dealing with this [42] the uncertainty around the estimate of cost-effectiveness was explored through multiple replication bootstrapping analyses, and visually represented using cost-effectiveness planes. Further studies examining the applicability and cost-effectiveness of such an intervention on a large scale population are warranted. Third, although accelerometers are more valid than self-report measures for measuring PA change they may induce a Hawthorn effect (i.e., an alteration of behaviour due to the awareness of being observed) [43]. Any accelerometer measurement reactivity should have been equal within groups at both baseline and follow-up and would not influence the measured change between the groups. In addition, Ullrich and colleagues found that in an adult population (65% women, mean age  =  54.6 y) all 7-day accelerometry assessments did not change MVPA levels [44]. Fourth, we recruited patients from an ambulatory secondary care hospital clinic. Patients attending ambulatory hospital clinics have higher rates of chronic disease than the general population and recruiting from one site only may impact the broad generalizability of the findings to other populations [45]. The average attainment of 22 ± 10 min of MVPA/ day at the 9-month follow-up in the intervention group demonstrate that the PA coaching intervention offers a low cost option for increasing PA in populations that may benefit from health behaviour change. Larger scale studies may permit subgroup-analyses to examine the cost-effectiveness of telephone coaching for patients with specific health conditions. Finally, the 9-month follow-up in this study, which included a 6-month non-intervention period, is sufficient to meet conventional standards of behaviour change maintenance [6]. Future research with a follow-up of more than 1 year is needed to investigate the long-term health and economic benefits of this PA telephone coaching.

## Conclusion

The Healthy 4U-2 programme is a low-cost strategy for increasing PA and QALYS among insufficiently physically active ambulatory hospital patients recommended to engage in the telephone coaching by consulting surgeons. The PA telephone coaching increased device-measured PA and quality of life and resulted in lower overall costs, mainly attributable to decreased healthcare costs. Findings of health benefits and cost-savings are infrequent and integrating PA telephone coaching into ambulatory hospital care offers a potentially cost-effective investment to produce better public health outcomes.

## Supporting information

S1 Checklist CHEERS checklist. (PDF)

S2 Checklist CONSORT checklist. (DOC)

S1 File Study protocol. (PDF)

S1 Table Unit pricing for subcategories of healthcare use. (DOCX)

S1 Fig Cost-effectiveness acceptability curves. (DOCX)

## References

[1] Warburton DR, Nicol CW, Bredin SD. Health benefits of physical activity: the evidence. CMAJ. 2006;174(6):801–9. doi: 10.1503/cmaj.051351 16534088 PMC1402378

[2] Lee IM, Shiroma EJ, Lobelo F, Puska P, Blair SN, Katzmarzyk PT, et al. Effect of physical inactivity on major non-communicable diseases worldwide: an analysis of burden of disease and life expectancy. Lancet. 2012;380(9838):219–29. doi: 10.1016/S0140-6736(12)61031-9 22818936 PMC3645500

[3] Ding D, Lawson KD, Kolbe-Alexander TL, Finkelstein EA, Katzmarzyk PT, Van Mechelen W, et al. The economic burden of physical inactivity: a global analysis of major non-communicable diseases. Lancet. 2016;388(10051):1311–24. doi: 10.1016/S0140-6736(16)30383-X 27475266

[4] Ding D, Kolbe-Alexander T, Nguyen B, Katzmarzyk PT, Pratt M, Lawson KD. The economic burden of physical inactivity: a systematic review and critical appraisal. BJSM. 2017;51(19):1392–409. doi: 10.1136/bjsports-2016-097385 28446455

[5] Foster C., Richards J., Thorogood M. and Hillsdon M., 2013. Remote and web 2.0 interventions for promoting physical activity. Cochrane Database of Systematic Reviews, (9). 10.1002/14651858.CD010395.pub2 PMC9674455 24085594

[6] Howlett N, Trivedi D, Troop NA, Chater AM. Are physical activity interventions for healthy inactive adults effective in promoting behavior change and maintenance, and which behavior change techniques are effective? A systematic review and meta-analysis. Trans Behav Med. 2019 Feb;9(1):147–57. 10.1093/tbm/iby010 PMC6305562 29506209

[7] Sparling PB, Owen N, Lambert EV, Haskell WL. Promoting physical activity: the new imperative for public health. Health Edu Res. 2000;15(3):367–76. doi: 10.1093/her/15.3.367 10977383

[8] Börjesson M. Promotion of physical activity in the hospital setting. Dtsch Z Sportmed. 2013;64:162–5.

[9] Allender S, Hutchinson L, Foster C. Life-change events and participation in physical activity: a systematic review. Health Prom Int. 2008;23(2):160–72. doi: 10.1093/heapro/dan012 18364364

[10] Thompson WR, Sallis R, Joy E, Jaworski CA, Stuhr RM, Trilk JL. Exercise is medicine. Am J Lifestyle Med. 2020;14(5):511–23. doi: 10.1177/1559827620912192 32922236 PMC7444006

[11] Barrett S, Begg S, O’Halloran P, Kingsley M. A physical activity coaching intervention can improve and maintain physical activity and health-related outcomes in adult ambulatory hospital patients: The Healthy4U-2 randomised controlled trial. Int J Behav Nut Phys Act. 2020;17(1):1–11. doi: 10.1186/s12966-020-01063-x 33256753 PMC7708221

[12] Barrett S, Begg S, Kingsley M. The Effect of a Physical Activity Coaching Intervention on Accelerometer-Measured Sedentary Behaviours in Insufficiently Physically Active Ambulatory Hospital Patients. Int Jour Enviro Res Pub Health. 2021;18(11):5543. doi: 10.3390/ijerph18115543 34067292 PMC8196832

[13] Mattli R, Farcher R, Syleouni M-E, Wieser S, Probst-Hensch N, Schmidt-Trucksäss A, et al. Physical activity interventions for primary prevention in adults: a systematic review of randomized controlled trial-based economic evaluations. Sports Medicine. 2020;50(4):731–50. doi: 10.1007/s40279-019-01233-3 31755043

[14] Drummond MF, Sculpher MJ, Claxton K, Stoddart GL, Torrance GW. Methods for the economic evaluation of health care programmes: Oxford university press; 2015.

[15] Husereau D, Drummond M, Petrou S, Carswell C, Moher D, Greenberg D, et al. Consolidated health economic evaluation reporting standards (CHEERS) statement. Int J Technol Assess Health Care. 2013;29(2):117–22. doi: 10.1017/S0266462313000160 23587340

[16] Fortier MS, Duda JL, Guerin E, Teixeira PJ. Promoting physical activity: development and testing of self-determination theory-based interventions. Int J Behav Nut Phys Act. 2012;9(1):1–14. doi: 10.1186/1479-5868-9-20 22385751 PMC3353256

[17] Barrett S, Begg S, O’Halloran P, Kingsley M. Integrated motivational interviewing and cognitive behaviour therapy can increase physical activity and improve health of adult ambulatory care patients in a regional hospital: the Healthy4U randomised controlled trial. BMC Public Health. 2018;18(1):1–11. 10.1186/s12889-018-6064-7 PMC6180400 30305078

[18] Freedson PS, Melanson E, Sirard J. Calibration of the computer science and applications, inc. accelerometer. Med Sci Sports Ex. 1998;30(5):777–81. doi: 10.1097/00005768-199805000-00021 9588623

[19] Nelson ME, Rejeski WJ, Blair SN, Duncan PW, Judge JO, King AC, et al. Physical activity and public health in older adults: recommendation from the American College of Sports Medicine and the American Heart Association. Circulation. 2007;116(9):1094. doi: 10.1161/CIRCULATIONAHA.107.185650 17671236

[20] Ware JE Jr, Kosinski M, Keller SD. A 12-Item Short-Form Health Survey: construction of scales and preliminary tests of reliability and validity. Medical Care. 1996:220–33. doi: 10.1097/00005650-199603000-00003 8628042

[21] Brazier J, Roberts J, Deverill M. The estimation of a preference-based measure of health from the SF-36. Jour Health Eco. 2002;21(2):271–92. doi: 10.1016/s0167-6296(01)00130-8 11939242

[22] FairWork Commission Australia. Allied Health Professionals (VICTORIAN PUBLIC HEALTH SECTOR) Single Interest Enterprise Agreement 2016–2020 [Available from: https://www.fwc.gov.au/documents/documents/agreements/fwa/ae424114.pdf

[23] Independent Hospital Pricing Authority. The Pricing Framework for Australian Public Hospital Services 2019–20. Available from: https://www.ihpa.gov.au/.

[24] Appleby J, Devlin N, Parkin D. NICE’s cost effectiveness threshold. British Medical Journal Publishing Group; 2007.

[25] Golsteijn RHJ, Peels DA, Evers SMAA, Bolman C, Mudde AN, de Vries H, et al. Cost-effectiveness and cost-utility of a Web-based or print-delivered tailored intervention to promote physical activity among adults aged over fifty: an economic evaluation of the Active Plus intervention. Int J Behav Nut Phys Act. 2014;11(1):1–17. doi: 10.1186/s12966-014-0122-z 25262435 PMC4189727

[26] Schilling C, Dowsey MM, Clarke PM, Choong PF. Using patient-reported outcomes for economic evaluation: getting the timing right. Value Health. 2016;19(8):945–50. doi: 10.1016/j.jval.2016.05.014 27987644

[27] Gray AM, Clarke PM, Wolstenholme JL, Wordsworth S. Applied methods of cost-effectiveness analysis in healthcare. OUP Oxford; 2010.

[28] Sevick MA, Dunn AL, Morrow MS, Marcus BH, Chen GJ, Blair SN. Cost-effectiveness of lifestyle and structured exercise interventions in sedentary adults: results of project ACTIVE. Am J Prev Med. 2000;19(1):1–8. doi: 10.1016/s0749-3797(00)00154-9 10865157

[29] Elley R, Kerse N, Arroll B, Swinburn B, Ashton T, Robinson E. Cost-effectiveness of physical activity counselling in general practice. New Zealand Medical Journal. 2004;117(1207):1–15. 15608809

[30] Harris T, Kerry S, Victor C, Iliffe S, Ussher M, Fox-Rushby J, et al. A pedometer-based walking intervention in 45-to 75-year-olds, with and without practice nurse support: the PACE-UP three-arm cluster RCT. Health Technol Assess. 2018;22(37):1–274. doi: 10.3310/hta22370 29961442 PMC6046648

[31] Holman HR. The relation of the chronic disease epidemic to the health care crisis. ACR Open Rheumatology. 2020;2(3):167–73. doi: 10.1002/acr2.11114 32073759 PMC7077778

[32] Martinson BC, Crain AL, Pronk NP, O’connor PJ, Maciosek MV. Changes in physical activity and short-term changes in health care charges: a prospective cohort study of older adults. Prev Med. 2003;37(4):319–26. doi: 10.1016/s0091-7435(03)00139-7 14507488

[33] Wu S, Cohen D, Shi Y, Pearson M, Sturm R. Economic analysis of physical activity interventions. Am J Prev Med. 2011;40(2):149–58. doi: 10.1016/j.amepre.2010.10.029 21238863 PMC3085087

[34] Woodcock J, Franco OH, Orsini N, Roberts I. Non-vigorous physical activity and all-cause mortality: systematic review and meta-analysis of cohort studies. Int J Epidemiol. 2011;40(1):121–38. doi: 10.1093/ije/dyq104 20630992

[35] Sevick MA, Napolitano MA, Papandonatos GD, Gordon AJ, Reiser LM, Marcus BH. Cost-effectiveness of alternative approaches for motivating activity in sedentary adults: results of Project STRIDE. Prev Med. 2007;45(1):54–61. doi: 10.1016/j.ypmed.2007.04.008 17573103 PMC2762938

[36] Ewald B, Stacey F, Johnson N, Plotnikoff RC, Holliday E, Brown W, et al. Physical activity coaching by Australian Exercise Physiologists is cost effective for patients referred from general practice. Aus New Zeal Jour Pub Health. 2018;42(1):12–5. doi: 10.1111/1753-6405.12733 29165855

[37] van Keulen HM, Bosmans JE, van Tulder MW, Severens JL, de Vries H, Brug J, et al. Cost-effectiveness of tailored print communication, telephone motivational interviewing, and a combination of the two: results of an economic evaluation alongside the Vitalum randomized controlled trial. Int J Behav Nut Phys Act. 2010;7(1):1–12. 10.1186/1479-5868-7-64 PMC2940922 20815869

[38] Goyder E, Hind D, Breckon J, Dimairo M, Minton J, Everson-Hock E, et al. A randomised controlled trial and cost-effectiveness evaluation of’booster’interventions to sustain increases in physical activity in middle-aged adults in deprived urban neighbourhoods. Health Technology Assessment. 2014;18(13):1. doi: 10.3310/hta18130 24571932 PMC4781193

[39] Oksman E, Linna M, Hörhammer I, Lammintakanen J, Talja M. Cost-effectiveness analysis for a tele-based health coaching program for chronic disease in primary care. BMC Health Serv Res. 2017;17(1):1–7. 28202032 10.1186/s12913-017-2088-4 PMC5312514

[40] Cutler DM. Behavioral health interventions: what works and why. Critical perspectives on racial and ethnic differences in health in late life. National Acadamies Press. 2004;643:674. 20669464

[41] Barrett S, Begg S, Sloane A, Kingsley M. Surgeons and preventive health: a mixed methods study of current practice, beliefs and attitudes influencing health promotion activities amongst public hospital surgeons. BMC Health Serv Res. 2019;19(1):1–12. 31170990 10.1186/s12913-019-4186-y PMC6555744

[42] Briggs AH. Handling uncertainty in cost-effectiveness models. Pharmacoeconomics. 2000 May;17(5):479–500. doi: 10.2165/00019053-200017050-00006 10977389

[43] Sedgwick P, Greenwood N. Understanding the Hawthorne effect. BMJ. 2015;351. doi: 10.1136/bmj.h4672 26341898

[44] Ullrich A, Baumann S, Voigt L, John U, Ulbricht S. Measurement Reactivity of Accelerometer-Based Sedentary Behavior and Physical Activity in 2 Assessment Periods. Journal of Physical Activity and Health. 2021 Jan 12;18(2):185–91. doi: 10.1123/jpah.2020-0331 33440344

[45] Steiner CA, Friedman B. Peer reviewed: Hospital utilization, costs, and mortality for adults with multiple chronic conditions, nationwide inpatient sample, 2009. Prev Chronic Disease. 2013;10. 10.5888/pcd10.120292 PMC3652722 23618542

