# Supplementary material for: Physical activity telephone coaching intervention for insufficiently physically active ambulatory hospital patients: Economic evaluation of the Healthy 4U-2 randomised controlled trial
Source: PLoS One. 2022 Jun 23;17(6):e0270211. doi: 10.1371/journal.pone.0270211 (PMC9223391; doi:10.1371/journal.pone.0270211)
Supplement: S1 File — (PDF) [file pone.0270211.s003.pdf]

Design and rationale of the Healthy 4U-2 trial of a telephone based intervention of a blend of motivational interviewing and cognitive behaviour treatment for physical activity behaviour change and maintenance: a randomised controlled trial protocol.

BACKGROUND

Non communicable diseases (NCDs) are the leading cause of illness, disability and death in Australia, accounting for 90% of all deaths in 2011 [1]. NCDs have an intricate association with modifiable risk factors such as physical inactivity, poor nutrition, and smoking [2]. Hospitals, as a result, have been required to broaden their role from their primary focus of disease treatment towards a position of more integrated health promotion and holistic care [5].

Hospitals are in a suitable position within the health care system to be advocates for health promotion [6]. Hospitals represent the primary concentration of health resources, professional skills and medical technology in the [5]. Moreover, hospital-based clinicians are seen as credible sources of advice and expertise on health issues that extend beyond their responsibilities for services related to sick care [7]. The extensive resources that hospitals command mean that even a small shift of focus has the potential to bring about an increase in resources dedicated to health promotion [5,6]. This shift, over time, could bring potential health benefits to a community [5, 7]. The hospital healthcare system also provides important opportunities to reach the most disadvantaged in the community, who are often hard to reach by wider population-based approaches [8].

Surgeons working in the hospital system have an important role in the promotion of healthy lifestyles and behaviour change for patients with, or at risk of, chronic disease [9]. Due to their extensive training, specialisation and medical expertise, surgeons are perceived as dependable

sources of advice and expertise on health issues that extend beyond their responsibilities for services related surgical care [6]. Surgeons as such, are influential in the promotion of lifestyle behaviour change [7]. Hospital surgeons also have significant influence over clinical settings, and by extension, institutional policy and practice [10]. The surgical profession is one of responsibility and leadership, playing key roles in strategic planning, especially pertaining to clinical care [10].

Patients have stated that they would like, and to some extent expect the healthcare system to provide some guidance on lifestyle behaviour change and physical activity (PA) [12]. Doctors practicing in hospitals have stated that they do not have sufficient time to spend with patients giving advice on preventive measures [13]. The working intensity of doctors make it difficult to spend sufficient time with patients discussing preventive health [13]. Unpublished data from Bendigo Health supports this, with doctors indicating that discussions about smoking, nutrition and physical activity (SNAP) health risk factors are very important for health, yet, at the same time they are not a high priority. This has resulted in low implementation rates of preventative health practices.

The H4U study demonstrated that integrated motivational interviewing (MI) and cognitive behaviour therapy (CBT) resulted in significant changes in physical activity and health related outcomes for secondary care participants. The H4U-2 extends the H4U study in a number of ways including: (i) increased focus of the intervention on maintenance of behaviour change; (ii) a longer follow-up timeframe to test the maintenance effect of the intervention; decrease in intervention number from 8 sessions to 5; and (iii) referrals by surgeons into the program. The H4U-2 offers surgeons a pathway to integrate preventive health into clinical care, delivering a brief intervention to at risk patients, and referring the patient in an efficient manner to a dedicated resource.

The primary objective of this study is to determine the effectiveness of lifestyle intervention in secondary care patients presenting to a non-admitted elective clinic in a public hospital who do not participate in enough physical activity to be deemed sufficiently physically active. Secondary aims relate to the effect of the MI/CBT intervention on self-efficacy, quality of life, and anthropometric measures. In addition, this study will investigate the acceptability, feasibility cost-effectiveness of delivering the telephone intervention in this setting.

METHODS AND ANALYSIS

Design

The H4U-2 project is a randomised controlled trial with assessment of outcomes at three points-baseline, post 12 weeks and 9 months designed and reported in line with the CONSORT recommendations for reporting (Figure 1). The study will be conducted at large regional tertiary public hospital that's serves a wide geographical region, with a socioeconomically diverse population. A total of 136 patients who present to BH Specialist Clinic as an elective admission will be recruited and included in the project (figure 1). The trial will be registered with the Australian and New Zealand Clinical Trials Registry prior to patient recruitment. The study will commence 01/12/18 and commence 01/12/22.

Randomisation

Participants who fulfil the inclusion criteria and consent to take part in the trial will randomised to either the intervention or the control group based on a random number sequence produced by a computer generated program (randomizer.org). Assignments will be prepared and sealed in sequentially numbered opaque envelopes. Assignment will be made by opening the next envelope in the sequence, after the recruiter determines eligibility for the study, participants

consent to take part, and baseline measurements are completed.

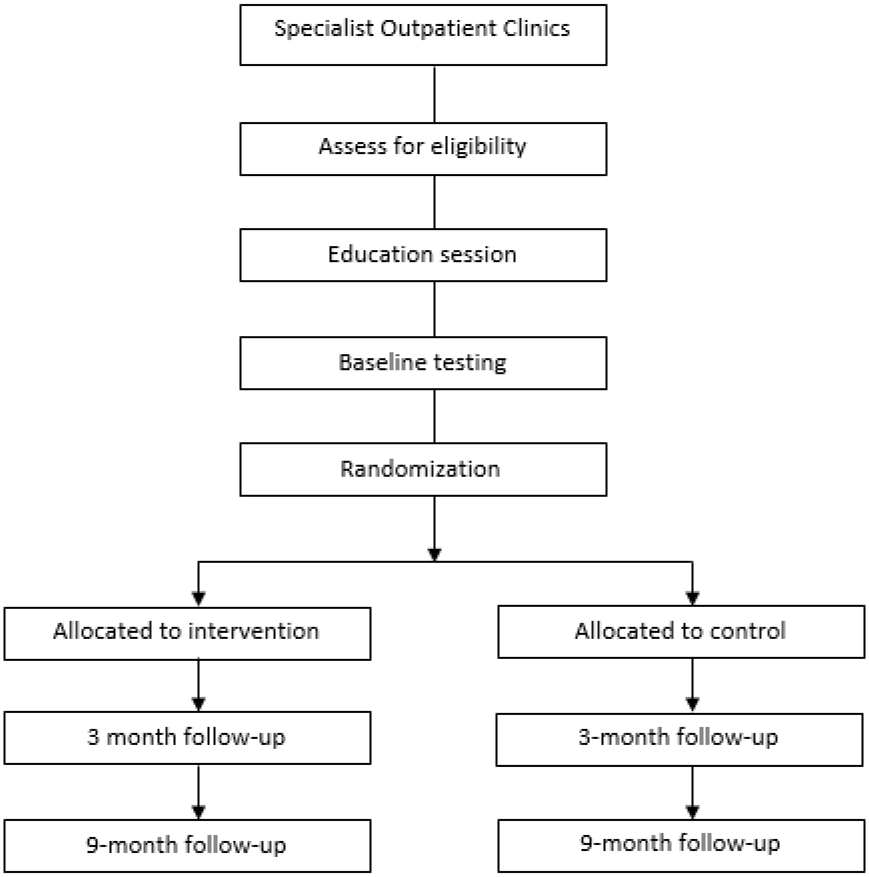

Figure 1: CONSORT Flow Chart

Study population

Group 1

The project target population is patients presenting to the BH Specialist Outpatients Clinic over a 12 month recruiting period. These patients are elective non-emergency presentations for a consultant with a medical specialist. Patients will be eligible if they are fluent in conversational English and insufficiently physically active (not participating thirty minutes or more of moderate

physical activity on at least five days a week- 150 mins/week) [10]. The exclusion criteria are: under 18 years and over 69 years; poor comprehension of English language (determined by project team); too physically active (participating in more than thirty minutes of moderate physical activity on at least five days a week); deaf/hearing impaired; disabling neurological disorder; severe mental illness such as psychosis, learning disability, dementia and cognitive impairment; registered blind; housebound or resident in nursing home; unable to move about independently or not ambulatory; pregnancy; advanced cancer. For those people who are ineligible or decline to participate, we will keep a 'screening log' of basic demographic information and reasons for non-participation.

Group 2 Participants as medical record audits.

A comparison cohort of patients attending Specialist Clinics will be selected for audit of healthcare usage over the study period. The Group 2 participants will be drawn from the Specialist Clinic client management records by Specialist Clinic administration staff. All the data will be retained in a study location on the password protected Bendigo Health corporate drives. The data collection timeframe is 1 September 2018 to 1 Dec, 2020.

Recruitment

All participants will be recruited from the BH Specialist Outpatients Clinic following the same pathway (figure 1):

- During the recruitment time-frame participating consultants will be provided with sequentially numbered invitational fliers to be distributed to potential participants. The fliers invite patients to participate in the project (appendix 1). The information flier will briefly detail to the patients what the project will entail and the exclusion criteria. This

pathway is an important step in the project design- clinicians who have a relationship with the individual may be in a good position to highlight the project, and its potential significance to the individual. This referral pathway is has the potential to be more sustainable in the long term aim of integrating health promotion into existing practice.

- A project team member will speak to all potential participants and provide details of the project. Potentially interested participants will be given the Patient Information Sheet and Consent (PICF)- the PICF form gives an in-depth synopsis of the project (appendix 2). A project team member will go through the project details and the PICF with any interested person. Potentially interested participants will also be given a copy of the PAR-Q which needs to be competed as part of inclusion criteria.

- To limit pressure and coercion on the individual, the PICF does not need to be signed and completed at that time. The participant may take the PICF home to read and discuss with others. Completed PICF's will be collected at the briefing sessions. Participants will be given a stamped addressed envelope which they can return the PICF to the project team should they wish.

- Participants who express an interest in participation but want to take time to consider their choice will be offered a follow-up phone call from the project team to further discuss the project. A project team member will take the participants phone number and discuss appropriate times to contact them.

- Follow-up calls to potential participants will follow the same structure:

    - A member of the project team will make all follow-up calls.

    - Potential participants will be contacted on the number that they supply to the project team.

    - Potential participants will be informed that they will be contacted no sooner than one week after talking to the project team member. This time will allow the individual time to consider their decision.

    - Should the project team member call and the individual does not answer, no message will be left. The project team member will make two attempts to contact on this occasion. If no contact is made no message will be left on this occasion.

In this circumstance the project team member will try to contact the individual again, a minimum of four days later. Two calls will be attempted, with a voice message left should the second attempt be unsuccessful. The message left will be scripted for uniformity:

"*Hello [potential participant], this is [project team member] from Bendigo Health calling you in regards to the Health 4U project that we discussed with you in the specialist outpatient clinic. This call is following up on our conversation about your potential interest in participating in the project. If you would like to discuss this further please contact the Healthy 4U team directly on 5454 9118. Thank you".*

- After leaving a voice message one more attempt at contact will be made, a minimum of four days later. If this call is unsuccessful no message will be left at this time, and no further contact will be attempted.

- If, on speaking to a potential participant the individual states that they are no longer interested in participating in the project the project officer will thank the individual for their time and consideration, and inform the individual that no further contact will be instigated by the project team.

- If, on speaking to a potential participant the individual states that they are still unsure about whether they are interested in participating in the project the project officer will ask if the individual would like to be contacted again to follow this up. Subsequent calls will follow the structure as above.

- For individuals who choose to participate in the project, a project team member will book them into one of the mandatory briefing sessions. A number of sessions will be held to accommodate participant schedules, including day and evening sessions. During this phone call the project team member will:

    - Remind participants to bring their PICF to the briefing session.

    - Discuss availability of briefing session times and dates, and book the participant into the most suitable session.

    - Check that the potential participant has completed a PAR-Q and that they bring it to the briefing session.

- At the briefing session participants will complete their outcome measures (appendix 4). Completion of the outcome measures should take approximately fifteen minutes. Members of the project team can assist during this time. The outcome measures will be taken prior to any briefing interventions. Participants will be informed that repeat measures are required after week 12 and at 9 months.

- At the end of the briefing session, the participants will be allocated into their respective groups- group 1 or group 2. This allocation will be completed through a computer-generated program. The particulars of each group will be outlined below in this document.

- All participants will be asked to attend two follow-up sessions, one after the 12-week intervention and the other at 9 months. A number of sessions will be held to accommodate participant schedules, including day and evening sessions. During attendance at the sessions repeat outcome measures will be taken and accelerometers will be fitted again. As a direct consequence of the social distancing measures resulting from the Covid-19 pandemic, no group measurements will be undertaken from 31 March 2020. For the remaining participants, self-reported measures of mass and waist-circumference will be recorded. Accelerometers will be delivered to participants, and collected at a designated time in a sealed envelope with no individual contact required. In situations where participants reside in geographic locations that prohibit delivery, accelerometers will be posted to participants, with a stamped envelope to return the accelerometers. All accelerometers and bands will be cleaned in line with recommendations from the Department of Health and Human Services.

- All participants will be offered a survey to complete as part of process evaluation (appendix 5). This survey will be distributed at the follow-up session and can also be completed online via survey money, and/or mail out copies.

- Participants who withdraw from the study who wish that their data is not included in the analysis will be required to complete a withdrawal of consent form.

Interventions

Briefing Session

All project participants enrolling in the project will have to attend a briefing session (Figure 1).

The briefing session will be a facilitated learning session based around self-management and lifestyle modification. Education sessions and problem solving will be developed and carried out using the Self Determination Theory (SDT) framework [37]. This theory is used to support, educate and motivate participants around positive lifestyle choices, as well as the empowerment of individuals over their health care [37]. SDT reflects that an individual's on-going functioning is a product of a continuous interaction between cognitive, behavioural, and contextual factors, and more than a matter of education by itself [37]. Classroom learning for example is shaped by factors within the academic environment, but particularly by the reinforcements experienced by individuals and by others [37]. To adhere to this framework, participants will be encouraged to participate actively in the briefing sessions through group and task orientated learning, and education will be delivered through case studies to stimulate vicarious learning [37]. Participants will be exposed to real life scenarios of health risk modification and given brief tasks to stimulate this thought process. SDT has been chosen as the framework in which to deliver the briefing session as has been shown to complement MI and CBT integration [37]. The briefing sessions will be facilitated by a member of the project team who is registered healthcare practitioner through the Australian Health Practitioner Regulating Authority (AHPRA). The sessions will also include a guest speaker representing the community health programs.

Intervention

The intervention group will receive the blend of MI and CBT. This model of patient-centred self-management support, delivered as an office-based program through telephone calls has demonstrated successful outcomes for similar populations [19,22,38-9]. A member of the project

team will facilitate the MI/CBT intervention via the telephone. The individual who will deliver the telephone support is a registered healthcare practitioner through the Australian Health Practitioner Regulating Authority (APHRA). He has attended a 2-day workshop on MI and has received additional coaching from Dr. Paul O’Halloran, who is a nationally endorsed Health Psychologist and Sport and Exercise Psychologist who has been registered and practicing as a Psychologist since 1998. The individual who will deliver the intervention also delivered 144 hours of MI-CBT during the H4U trial.

Each participant will receive 5 phone calls, each lasting up to 20 minutes. Telephone support following a CBT approach, bedded in MI philosophy has shown to be effective in behaviour modification [38-9]. Participants will receive 4 calls over a 6-week period, with a ‘booster’ call at 12 weeks. In terms of incorporating the MI/CBT blend, MI will be utilised for sessions 1 to 3, as MI is identified as an effective tool for behaviour change initiation [21]. From session 3 to session 5 a blend of MI/CBT will be used, as CBT is an action orientated treatment [21, 23-4].

As part of self-management support, participants, where the intervention is appropriate, will be made aware of programs aimed at lifestyle risk modification existing in the community. Following the MI/CBT theory, no mandatory referrals will be made to these programs, however the facilitator may highlight the existence of these programs should the situation warrant it. The facilitators will be aware of the programs that are available in the community and can make appropriate referral to the programs on behalf of the participants. This pathway may highlight to participants the range of programs that are available in the community. Utilizing existing programs in the community may be of benefit to the participants, while it also encourages service co-ordination and avoidance of service duplication.

Control

All control group participants will attend the briefing session so they will receive the same educational material around lifestyle risk factor modification as group 1 from that session.

Participants of the control group will be contacted to enquire about their attendance at the follow-up session, reminding them about the requirement for outcome measure completion. Apart from this contact participants the control group will receive no further routine contact initiated by the project team.

Intervention Fidelity

To assist with improving the credibility of evidence resulting from behaviour change studies the reporting on treatment fidelity has been recommended [40]. Assessments in intervention fidelity are integral to intervention research but few published trials report these processes in detail [41]. This methodological shortcoming makes it very difficult to distinguish between the quality of behaviour change interventions, and, consequently, to be able to establish whether MI provision has contributed to any intervention effects [41]. Treatment fidelity has been defined as the "methodological strategies used to monitor and enhance the reliability and validity of behavioural interventions" [41]. To measure the fidelity of the treatment the Motivational Interviewing Treatment Integrity code will be used [42].

For measurement of fidelity 10% of counselling sessions in the study will be digitally recorded. The recorded sessions will be assessed for adherence to MI principles using the Motivational Interviewing Treatment Integrity code [26] by an expert independent coding group. Coders are blind to the study arm of the session and the study hypotheses. We anticipate that MI sessions will receive scores of 4 or higher on the 1–5 global rating. With respect to frequency measures of counsellor behaviour we expect MI sessions to have a higher reflection to question ratio and significantly fewer instances of giving information.

Study Outcomes

The primary endpoint is a change in physical activity levels, measured at 3 points- baseline, 13-weeks, and at 9 months (Table 1). Secondary endpoints include a change in self-efficacy, quality of life, waist circumference, BMI, and smoking status (Table 1.)

Physical Activity

The primary endpoint is a change in physical activity levels. Physical activity will be measured objectively using an accelerometer (ActiGraph, Florida, United States) and a log diary to document the type of activity completed, and any occasions when the accelerometer was removed.

The ActiGraph GT3X+ is a validated, tri-axial activity monitor that provides data on physical activity including activity counts, steps and activity intensity (METs) [43]. The output from the tri-axial accelerometer measures time spent in physical activity using standard cut-off points for sedentary, light, moderate, vigorous and very vigorous physical activity. The primary outcome measure will be minutes of physical activity at moderate intensity or greater per week.

The research team will explain to the participant how to wear the accelerometer, requesting it to be worn from waking in the morning until going to bed at night. Participants will be asked to remove the ActiGraph when they are showering, bathing or swimming. After seven days of wearing the accelerometer, the accelerometers will be collected at the next possible visit to the hospital or arrangement will be made for collection from participants at a location most convenient to them.

Self-Efficacy

Self-Efficacy to be physically active will be measured via a physical activity self-efficacy survey. This survey has been built upon previous research, with modifications to suit the target group that this study proposes [44]. The survey measures confidence related to completing physical activity while faced with recognised barriers to physical activity completion.

Medical Outcomes Study Short Form 12 Health Survey (SF-12)

To measure quality of life the Medical Outcomes Study Short Form 12 Health Survey (SF-12) will be used [49]. The SF-12 was derived from twelve questions of the SF-36, which make up the MCS (Mental Component Summary) and PCS (Physical Component Summary). The survey can be administered in two to three minutes, which saves both time and resources in large-scale population surveys. The SF-36 has been validated in an Australian population 3. The two scores range between 0 and 100, with increasing values equating to better health.

Using the SF-12 also allows for conversion to utility scores which can also facilitate the undertaking of an economic analysis.

Table 1. Primary and secondary outcome measures

**Primary and secondary outcome measures**
Primary<br>• Physical Activity – ActiGraph [43]
Secondary<br>• Self-Efficacy- Modified physical activity self-efficacy survey [44]<br>• Quality of Life: SF-12 Survey [49]<br>• Waist Circumference- within AUSDRISK tool [50]<br>• Smoking- within AUSDRISK tool [50]<br>• Nutrition- within AUSDRISK tool [50]<br>• BMI

Sample Size and Statistical Analysis

To compare differences in our outcome measures a series of 3 x 2 mixed model ANOVAs will be undertaken. Utilizing data from the H4U study, to detect an effect size of 0.35 or greater, with the alpha set at .05, and the power set at .90, a sample size of 50 participants per arm will be required. Protecting against a drop-out rate of 20% over the 9 month period, 68 participants will be recruited and randomized into each arm. The study therefore aims to recruit approximately 136 participants.

A full intention-to-treat principle will be used. For participants with missing data at 3 month and 9 month the last-observation-carried forward approach will be adopted (Shao and Zhong, 2003). Repeat sensitivity analyses will be undertaken to provide indication that the imputed values have a significant effect on the outcome.

Process measures

The project team will log the number of referrals made by the surgeons in Specialist Clinic. The project team will record patient feedback regarding interest and opinions on the project, which is hoped may give insight into future program development strategies.

A log will be maintained of how many project participants contact the project team during the intervention phase, the reason for contact, the method of contact and the outcome. During any non-structured contact no intervention is to be delivered at that time.

To examine the acceptability of the H4U study, participants allocated to the intervention group will also be administered an additional questionnaire, after the 9 months follow up. This questionnaire will include items addressing satisfaction with the components of the project, their perceived utility of the intervention. The questionnaire will also address whether participants attended a community health program, and the frequency of visits. To obtain a more in-depth understanding of the potential barriers and facilitators at the service delivery and individual level to uptake of this programme, we will also conduct semi structured interviews with a subsample of participants in the intervention group. Recruitment for the interviews will be purposive to maximise variation such as urban/rural location, gender and age for patients. Sampling will continue until no new themes or categories emerge from the data (the so-called thematic saturation) [31]. The interviews will be conducted face-to-face or by telephone by a project officer with experience in qualitative interviews. Participants will be asked about their satisfaction with the program, whether they perceived it to be effective, what they liked or disliked, and their perception of the content. Interviews will be digitally recorded and transcribed. Interview transcripts and socio-demographic data will be entered as a project in NVIVO (QSR NVIVO7, Doncaster, Australia), a program for managing and analysing qualitative data. The

NVIVO software enables multilevel coding of text against a set of identified analytical categories. The study team will use an iterative process to understand the themes and key issues arising from the data.

Economic analysis

Changes to both health outcomes and to economic costs from a larger adoption of the supporting self-management project may be estimated with cost-effectiveness analysis. A health sector perspective will be adopted and values collected for variables that describe the participant's use of hospital health services during the timeframe of the study. This will include data from emergency presentation and inpatient admissions. These costs will be valued using market prices, or shadow prices will be imputed. The cost of delivering the H4U service will be assessed by measuring and valuing the resources used [53].The intervention costs will be calculated, factoring in staff time involved in being trained, and in delivering the interventions. Also included will be any overhead costs and costs of sessions provided. For the group intervention the costs will be divided over the attendees.

Changes to health benefits will be assessed through the use of quality-adjusted life-years. The SF-12 Health Survey can be converted to short form 6-dimension (SF-6D) which allow for utility values to be calculated [49]. To calculate the quality-adjusted life years (QALY) gain over the entire follow-up period an ‘area under the curve’ methodology will be used [53]. As costs are likely to be higher for one group compared to the other, and QALY gains may differ, an incremental cost-effectiveness ratio can be constructed to show the cost per extra QALY gained [53]. There will be uncertainty around cost and QALY estimates and this will be explored using cost-effectiveness planes generated from 1,000 bootstrapped resamples of the data for each of the three comparisons. Finally, we will generate cost-effectiveness acceptability curves, using the net-benefit approach and bootstrapping, to indicate the probability that any of the three approaches is the most cost-effective for different values placed on a QALY gain.

Within a project of this size significant differences in quality of life between the groups may not be seen, however, collecting data will provide an estimation of the baseline quality of life in this patient population. Cost-effectiveness acceptability curves can be plotted and used to inform decisions around widespread adoption of this method.

Longer terms costs and quality-adjusted life-years will be modelled using a decision analytic Markov model. The advantage of using the Markov model is that we can extrapolate beyond the data collection period and describe longer term costs and benefits of the intervention. While this method enables longer term costs and outcomes of the intervention to be forecast, the decision uncertainty will increase. To appropriately quantify uncertainty, Monte Carlo re-samples will be drawn from probability distributions specified for all model parameters.

Ethics and Dissemination

Following the statistical analysis reports will be produced and disseminated to management of 'Healthy Communities and Continuing Care' in Bendigo Health.

Results and findings may be presented at external conferences.

Results and findings may be detailed and submitted for publication in peer-reviewed journals.

CONCLUSION

The H4U-2 study will evaluate an innovative means of increasing physical activity in patients attending a non-admitted elective clinic in a public hospital who are deemed insufficiently physically active. The Healthy 4U-2 study builds upon the recommendations of the Healthy 4U study, incorporating a longer follow-up time to assess maintenance of behaviour change, as well as undertaking a more robust economic analysis. Due to the positive results of the Healthy 4U study the Healthy 4U-2 study will decrease the number of intervention sessions from 8 sessions to 5 sessions. The H4U-2 study has potential as a cost-effective home office based intervention

for physical activity uptake. The effectiveness of the intervention can be demonstrated in a rigorously conducted clinical trial.

References:

1. Australian Institute of Health and Welfare. Key indicators of progress for chronic disease and associated determinants: data report. Cat. no. PHE 142. [Internet]. Canberra: AIHW. 2011. Available from: http://www.aihw.gov.au/australias-health/2014/ill-health/#t2 (Accessed April 5, 2016)
2. World Health Organization. (2014) Global Status Report on Non-communicable diseases. [Internet] Available from: http://apps.who.int/iris/bitstream/10665/148114/1/9789241564854_eng.pdf?ua=1 (Accessed 5 April 2016)
3. Begg S, Vos T, Barker B, Stevenson C, Stanley L, Lopez AD. The burden of disease and injury in Australia 2003. Cat. no. PHE 82. [Internet]. Canberra: AIHW Available from: http://www.aihw.gov.au/publication-detail/?id=6442467990. (Accessed April 5, 2016)
4. Australian Bureau of Statistics. Key findings. In: Australian Health Survey: Updated Results, 2011-2012. Canberra: Australian Bureau of Statistics; 2013. [Internet] Canberra: AIHW. Available from: http://www.abs.gov.au/ausstats/abs@.nsf/Lookup/4364.0.55.003main+features12011-2012 (Accessed April 27 2016)
5. Community Profile. Profile.id. 2011. [Internet] Available from: http://profile.id.com.au/bendigo/seifa-disadvantage. (Accessed April 27 2016).
6. Department of Health and Human Services 2016, Victorian Population Health Survey 2014: Modifiable risk factors contributing to chronic disease, State Government of Victoria, Melbourne. [internet] Available from: https://www2.health.vic.gov.au/public-health/populationhealth-systems/health-status-of-victorians/survey-data-and-reports/victorian-population-health-survey.
7. Blair S, Kampert J, Kohl H, Barlow C, Macera C, Paffenbarger R, Gibbons L. Influences of cardiorespiratory fitness and other precursors on Cardiovascular Disease and all-cause mortality in men and women. JAMA. 1996; 276: 205–210. doi: 10.1001/jama.276.3.205
8. Beaglehole R, Bonita R, Horton R, Adams C, Alleyne G, Asaria P, et al. Priority actions for the non-communicable disease crisis. Lancet. 2011 Apr 23; 377(9775): 1438-47. doi: 10.1016/S0140-6736(11)60393-0. Epub 2011 Apr 5
9. Crespo C, Palmieri M, Perdomo R, McGee D, Smit E, Sempos C, et al. The relationship of physical activity and body weight with all-cause mortality: Results from the Puerto Rico heart health program. Annals of Epidemiology. 2002; 12: 543–552. doi: 10.1016/S1047-2797(01)00296-4.
10. Brown WJ, Bauman AE, Bull FC, Burton NW. Development of Evidence-based Physical Activity Recommendations for Adults (18-64 years). Report prepared for the Australian Government Department of Health, August 2012.
11. Blair S, Kampert J, Kohl H, Barlow C, Macera C, Paffenbarger R, Gibbons L. Influences of cardiorespiratory fitness and other precursors on Cardiovascular Disease and all-cause mortality in men and women. JAMA. 1996;276:205–210. doi: 10.1001/jama.276.3.205
12. Australian Bureau of Statistics. (2012). Australian Social Trends. [internet] Available from: http://www.abs.gov.au/AUSSTATS/abs@.nsf/allprimarymainfeatures/E0094FF470CFB2A0CA257A840015FA3D?opendocument (Accessed April 5, 2016)
13. Nigg, C. R., Borrelli, B., Maddock, J. and Dishman, R. K. (2008), A Theory of Physical Activity Maintenance. Applied Psychology: An International Review. 57: 544–560. doi: 10.1111/j.1464-0597.2008.00343.x
14. Bandura, A. (1997). Self-efficacy: The exercise of control. New York: Freeman.

15. McLeroy, K.R., Bibeau, D., Steckler, A., & Glanz, K. (1988). An ecological perspective on health promotion programs. Health Education Quarterly, 15, 351–377.
16. Latimer, A.E., & Ginis, K.A.M. (2005). Change in self-efficacy following a single strength training session predicts sedentary older adults' subsequent motivation to join a strength training program. American Journal of Health Promotion, 20, 135–138.
17. Naar-King, Sylvie; Earnshaw, Paul; Breckon, Jeff. Toward a Universal Maintenance Intervention: Integrating Cognitive Behavioral Treatment With Motivational Interviewing for Maintenance of Behavior Change. Journal of Cognitive Psychotherapy. Volume 27, Number 2; 126-137
18. Bennett JA, Lyons KS, Winters-Stone K, Nail LM, Scherer J: Motivational interviewing to increase physical activity in long-term cancer survivors. Nurs Res 2007, 56:18–27.
19. Hardcastle S, Taylor AH, Bailey M, Castle R: A randomised controlled trial on the effectiveness of a primary health care based counselling intervention on physical activity, diet and CHD risk factors. Patient Educ Couns 2008, 70:31–39.
20. Carels RA, Darby L, Cacciapaglia HM, Konrad Coit C, Harper J: Using motivational interviewing as a supplement to obesity treatment: A stepped-care approach. Health Psychol 2007, 26:369–374.
21. Miller WR, Rollnick S: Motivational interviewing: Preparing people for change. 2nd edition. New York: Guilford Press; 2002.
22. Hardcastle SJ. Effectiveness of a motivational interviewing intervention on weight loss, physical activity and cardiovascular disease risk factors: a randomised controlled trial with a 12-month post-intervention follow-up. International Journal of Behavioral Nutrition and Physical Activity 2013, 10:40
23. H.A. Flynn. Setting the stage for the integration of motivational interviewing with cognitive behavioral therapy in the treatment of depression. Cognitive and Behavioral Practice. 18 (2011), pp. 46–54
24. Hobbis ICA, Sutton S. Are techniques used in cognitive behaviour therapy applicable to behaviour change interventions based on the theory of planned behaviour? J Health Psychol. 2005;10(1):7–18. doi: 10.1177/1359105305048549.
25. Burke, B. L. (2011). What can motivational interviewing do for you? Cognitive and Behavioral Practice, 18, 74–81.
26. Moyers, T. B., & Houck, J. (2011). Combining motivational interviewing with cognitive-behavioral treatments for substance abuse: Lessons from the COMBINE research project. Cognitive and Behavioral Practice. 18, 38–45.
27. P.C. Britton, H. Patrick, A. Wenzel, G.C. Williams Integrating motivational interviewing and self-determination theory with cognitive behavioral therapy to prevent suicide. Cognitive and Behavioral Practice. 18 (2011), pp. 16–27
28. Greaves C, Sheppard K, Abraham C, Hardeman W, Roden M, Evans PH, et al. Systematic review of reviews of intervention components associated with increased effectiveness in dietary and physical activity interventions. BMC Public Health. 2011;11:119. doi: 10.1186/1471-2458-11-119.
29. National Collaborating Centre for Methods and Tools (2011). Appraising public health interventions. Hamilton, ON: McMaster University. (Updated 02 November, 2011) Retrieved from http://www.nccmt.ca/resources/search/101.
30. Beswick AD, Rees K, Griebsch I, et al. Provision, uptake and cost of cardiac rehabilitation programmes: improving services to under-represented groups. Health Technology Assessment 2004;8:1-15
31. Briffa TG, Kinsman L, Maiorana AJ, et al. An integrated and coordinated approach to preventing recurrent coronary heart disease events in Australia: a policy statement from the Australian Cardiovascular Health and Rehabilitation Association. Med J Aust 2009;190:683–68

32. Valkenet K, van de Port IG, Dronkers JJ, de Vries WR, Lindeman E, Backx F. The effects of preoperative exercise therapy on postoperative outcome: a systematic review. Jour Clin Rehabil. 2011 Feb;25(2):99-111.
33. O'Halloran P, Blackstock F, Shields N, et al. Motivational interviewing to increase physical activity in people with chronic health conditions: A systematic review and meta-analysis. Clin Rehabil 2014; 28(12): 1159–1171.
34. Thomas S, Reading J, Shephard RJ. Revision of the Physical Activity Readiness Questionnaire (PAR-Q). Can J Sport Sci. 1992 Dec;17(4):338-45.
35. Bendigo Health Care Group. Strategic Plan 2013-2016. Available from: http://bstrat2.grindstone.com.au/. (Accessed April 6, 2016).
36. John S Humphreys, John Wakerman, Robert Wells, Pim Kuipers, Judith A Jones and Phil Entitle. Beyond workforce": a systemic solution for health service provision in small rural and remote communities. Med J Aust 2008; 188 (8 Suppl): S77.
37. Patrick H, Williams G. Self-determination theory: its application to health behavior and complementarity with motivational interviewing. IJBNPA. 2012;9(18):1–12.
38. Burke L, H Lee, Jonine Jancey, Liming Xiang, Deborah A Kerr, Peter A Howat, et al. Physical activity and nutrition behavioural outcomes of a home-based intervention program for seniors: a randomized controlled project. International Journal of Behavioral Nutrition and Physical Activity 201310:14
39. Blackford K, Jancey J, Lee A, James A, Howat P, Waddell T. Effects of a home-based intervention on diet and physical activity behaviours for rural adults with or at risk of metabolic syndrome: a randomised controlled project. International Journal of Behavioral Nutrition and Physical Activity. 201613:13 DOI: 10.1186/s12966-016-0337-2
40. Jelsma, Judith G.M. et al. How to Measure Motivational Interviewing Fidelity in Randomized Controlled Trials: Practical Recommendations. Contemporary Clinical Trials 2015, Volume 43 ,93 – 9
41. Bellg AJ, Borrelli B, Resnick B, Hecht J, Minicucci DS, Ory M et al. Enhancing treatment fidelity in health behavior change studies: best practices and recommendations from the NIH Behavior Change Consortium. Health Psychol. 2004 Sep; 23(5):443-51.
42. Miller WR, Rollnick S. The effectiveness and ineffectiveness of complex behavioral interventions: impact of treatment fidelity. Contemporary clinical trials. 2014 Mar 31;37(2):234-41.
43. Plasqui G, Westerterp K. Physical activity assessment with accelerometers: an evaluation against doubly labeled water. Obesity (Silver Spring). 2007;15:2371–9. doi: 10.1038/oby.2007.28
44. Sallis, J.F., Pinski, R.B., Grossman, R.M., Patterson, T.L., and Nader, P.R. (1988). The development of self-efficacy scales for health-related diet and exercise behaviors. Health Education Research. 3, 283-292.
45. Blair SN, Dunn AL, Marcus BH, Carpenter RA, Jaret P: Active living everyday: 20 weeks to lifelong vitality. 2001, Champaign, Illinois: Human Kinetics
46. Prochaska JO, DiClemente CC, Norcross J. In search of how people change. American Psychologist. 1992;47:1102–14.
47. Marcus BH, Owen N. Motivational readiness, self-efficacy and decision-making for exercise. J Appl Soc Psychol. 1992;22:3–16.
48. Gawwad, Ensaf S.A. "STAGES OF CHANGE IN PHYSICAL ACTIVITY, SELF EFFICACY AND DECISIONAL BALANCE AMONG SAUDI UNIVERSITY STUDENTS." Journal of Family & Community Medicine 15.3 (2008): 107–115.
49. Brazier J. Roberts, J. The Estimation of a Preference-Based Measure of Health from the SF-12. Medical Care. 2004 September; 42(9):851-859
50. Chen L, Magliano DJ, Balkau B, Colagiuri S, Zimmet PZ, Tonkin AM, Mitchell P, Phillips PJ, Shaw JE. AUSDRISK: an Australian Type 2 Diabetes Risk Assessment Tool based

on demographic, lifestyle and simple anthropometric measures. Med J Aust. 2010 Feb 15;192(4):197-202.

51. Wilcox S, Dowda M, Leviton LC, Bartlett-Prescott J, Bazzarre T, Campbell-Voytal K, Carpenter RC, Castro CM, Dowdy D, Dunn AL, Griffin SF, Guerra M, King AC, Ory MG, Rheaume C, Tobnick J, Wegley S. Active for Life: Final Results from the Translation of Two Physical Activity Programs. American Journal of Preventive Medicine. 2008;35(4):340–351. doi: 10.1016/j.amepre.2008.07.00
52. Castro CM, Pruitt LA, Buman MP, King AC. Physical Activity Program Delivery by Professionals versus Volunteers: the TEAM Randomized Trial. Health psychology : official journal of the Division of Health Psychology, American Psychological Association. 2011;30(3):285-294. doi:10.1037/a0021980.
53. Drummond MF, Sculpher MJ, Torrance GW, et al. Methods for the Economic Evaluation of Health Care Programmes. 3rd edn. Oxford: Oxford University Press, 2005
