# Supplementary material for: Physical activity telephone coaching intervention for insufficiently physically active ambulatory hospital patients: Economic evaluation of the Healthy 4U-2 randomised controlled trial
Source: PLoS One. 2022 Jun 23;17(6):e0270211. doi: 10.1371/journal.pone.0270211 (PMC9223391; doi:10.1371/journal.pone.0270211)
Supplement: S1 Table — (DOCX) [file pone.0270211.s004.docx]

S4 Table. Unit pricing for subcategories of healthcare use

| **Cost category** | **Unit cost price AU$ ^a^** |
| --- | --- |
|  |  |
| **Allied Health** |  |
| Hydrotherapy | 193 |
| Physiotherapy | 149 |
| Psychology | 289 |
| Dietetics | 170 |
| Occupational Therapy | 180 |
| Podiatry | 191 |
|  |  |
| **Emergency Care** |  |
| General Injury | 541 |
| Gastrointestinal complaint | 386 |
| Musculoskeletal complaint | 483 |
|  |  |
| **Medical Specialist** |  |
| Endocrinology | 208 |
| Orthopaedics | 230 |
| Gynaecology | 264 |
| General medicine | 341 |
| General surgery | 265 |

^a^Unit cost prices are indexed to 2019 costs, based on Australian pricing guidelines
