# Supplementary material for: Physical activity telephone coaching intervention for insufficiently physically active ambulatory hospital patients: Economic evaluation of the Healthy 4U-2 randomised controlled trial
Source: PLoS One. 2022 Jun 23;17(6):e0270211. doi: 10.1371/journal.pone.0270211 (PMC9223391; doi:10.1371/journal.pone.0270211)
Supplement: S1 Fig — (DOCX) [file pone.0270211.s005.docx]

**S5 Fig. Cost-effectiveness acceptability curves.**


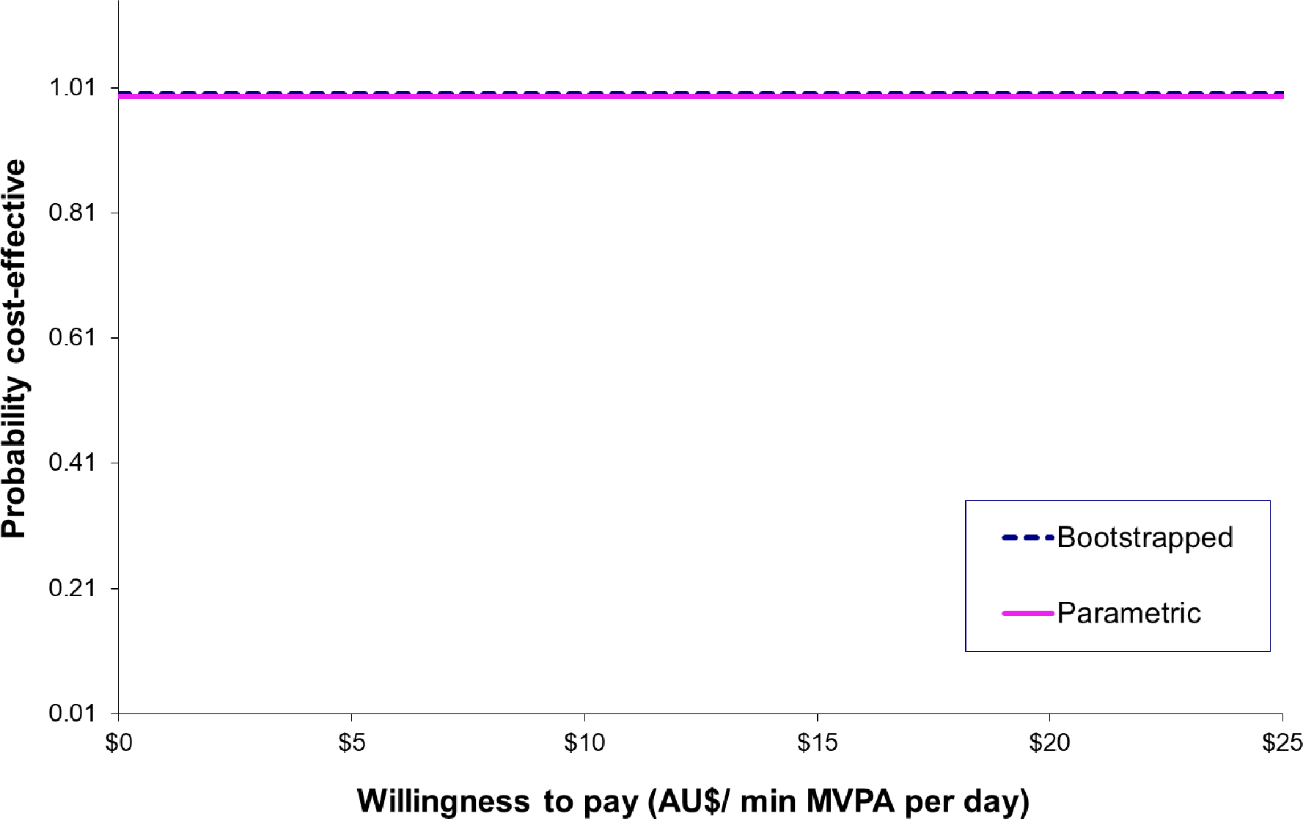


Cost-effectiveness acceptability curve showing the probability of the interventions being cost-effective in comparison to control for moderate-to-vigorous physical activity (MVPA).


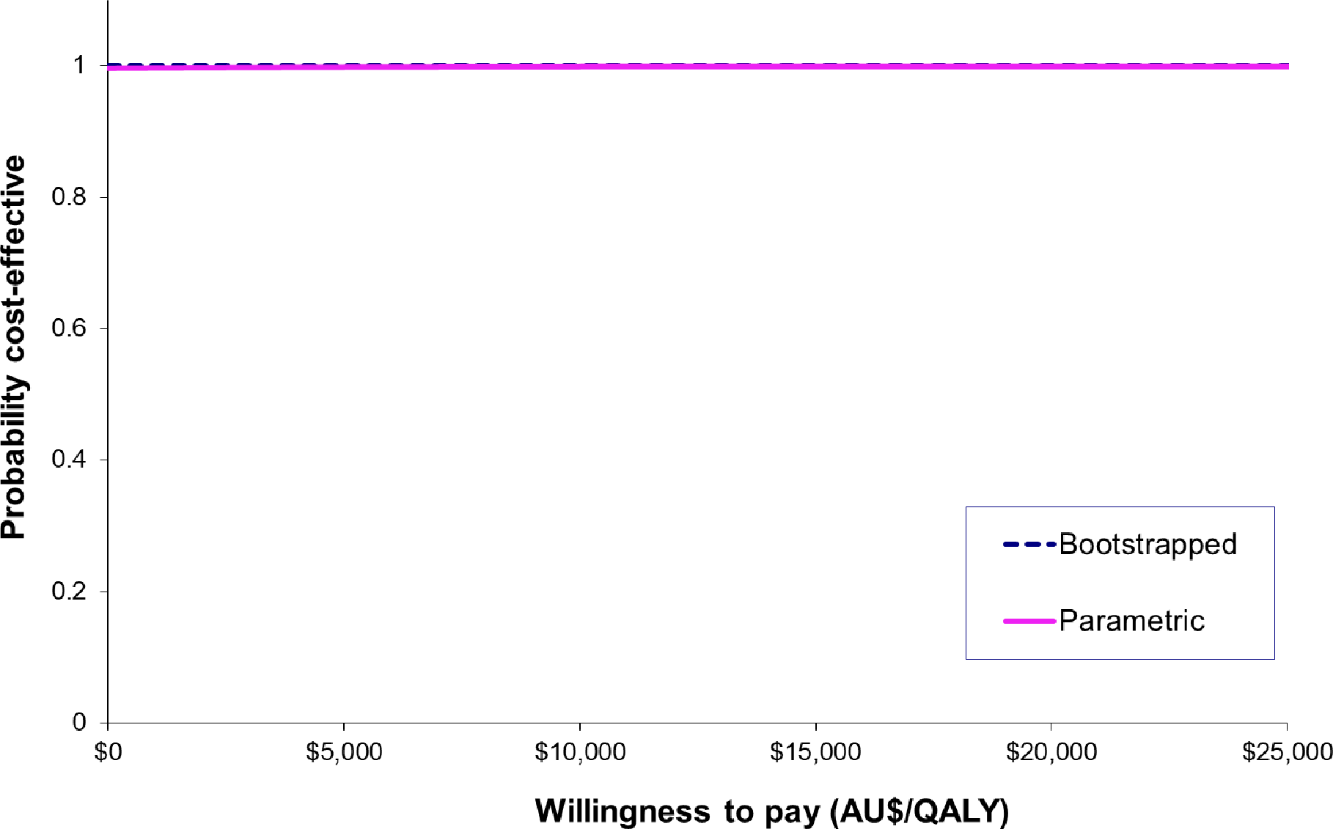


Cost-effectiveness acceptability curve showing the probability of the interventions being cost-effective in comparison to control for quality-adjusted life-years (QALYs).
